# Symptom burden in young adult brain tumor survivors: Key intervention targets based on patient-reported outcome network analyses

**DOI:** 10.1093/nop/npag015

**Published:** 2026-02-25

**Authors:** Charlotte Sleurs, Floortje Mols, Laurien De Roeck, Rhodé M Bijlsma, Suzanne E J Kaal, Jacqueline M Tromp, Monique E M M Bos, Tom van der Hulle, Ann Hoeben, Janine Nuver, Mathilde C M Kouwenhoven, Winette T A van der Graaf, Olga Husson

**Affiliations:** Department of Cognitive Neuropsychology, Tilburg University, Tilburg, The Netherlands (C.S.); Department of Oncology, KU Leuven, Leuven, Belgium (C.S., L.D.R.); CoRPS—Center of Research on Psychological disorders and Somatic diseases, Department of Medical and Clinical Psychology, Tilburg University, Tilburg, The Netherlands (F.M.); Department of Research & Development, Netherlands Comprehensive Cancer Organisation (IKNL), Utrecht, The Netherlands (F.M.); Department of Oncology, KU Leuven, Leuven, Belgium (C.S., L.D.R.); Department of Medical Oncology, University Medical Center Utrecht, Utrecht, The Netherlands; Department of Medical Oncology, Radboud University Medical Center, Nijmegen, The Netherlands (S.E.J.K.); Department of Medical Oncology, Amsterdam University Medical Centers, Amsterdam, The Netherlands (J.M.T.); Department of Medical Oncology, Erasmus MC Cancer Institute, Erasmus University Medical Center, Rotterdam, the Netherlands (M.E.M.M.B.); Department of Medical Oncology, Leiden University Medical Center, Leiden, The Netherlands; Department of Medical Oncology, GROW School for Oncology and Reproduction, Maastricht University Medical Centre, Maastricht, The Netherlands (A.H.); Department of Medical Oncology, University Medical Center Groningen, Groningen, The Netherlands (J.N.); Department of Neurology, Amsterdam UMC, The Netherlands (M.C.M.K.); Cancer Center Amsterdam, Treatment and Quality of Life, Amsterdam, The Netherlands (M.C.M.K.); Department of Medical Oncology, Leiden University Medical Center, Leiden, The Netherlands; Department of Medical Oncology, Netherlands Cancer Institute—Antoni van Leeuwenhoek, Amsterdam, the Netherlands; Department of Medical Oncology, Netherlands Cancer Institute—Antoni van Leeuwenhoek, Amsterdam, the Netherlands; Department of Surgical Oncology, Erasmus MC Cancer Institute, Erasmus University Medical Center, Rotterdam, the Netherlands (O.H.); Department of Public Health, Erasmus MC Cancer Institute, Erasmus University Medical Centre, Rotterdam, The Netherlands (O.H.)

**Keywords:** patient-reported outcomes, rehabilitation targets, symptom network modeling, treatment-related late effects

## Abstract

**Background:**

A brain tumor can lead to functional impairment, which is particularly concerning for adolescents and young adults (AYA). Patient-reported outcomes (PROs) have typically been examined as isolated domains, rather than as covarying symptoms. This study modeled PRO networks, symptom clustering, and topology among AYA oncology survivors.

**Methods:**

Patient-reported outcome networks from 4005 survivors were compared in topology between survivors of primary CNS tumors (n = 164) and non-CNS tumors (n = 3841). Survivors were diagnosed between 1999 and 2015 at ages 18 to 39 years, who completed the EORTC QLQ-SURV100 (Mdn follow-up = 12.31 years). Group-specific networks were estimated based on 33 health-related quality of life (HRQoL) scale scores using graphical LASSO. Wilcoxon rank-sum tests and the Network Comparison Test assessed group differences in the original PRO scales and their network centrality, respectively. Within the CNS subgroup (n = 164), associations with tumor-related and treatment-related characteristics were explored.

**Results:**

Survivors of central nervous system (CNS) tumors reported higher symptom burden on most PRO scales, along with a more diffuse network showing weaker within-domain cohesion (lower nodal strength and expected influence) and limited cross-domain integration (lower bridge strength). A small subset of nodes showed higher bridge expected influence (ie, fatigue, physical functioning, sexual problems when sexually active, work), which may represent key targets for intervention. Across both groups, negative health outlook, health distress, and physical functioning emerged as consistent core targets.

**Conclusion:**

Core symptoms may warrant prioritization in clinical follow-up and treatment of cancer survivors. These findings contribute to further development and optimization of tailored neurorehabilitation programs in neuro-oncological care.

Key Points

- CNS tumor survivors showed higher symptom burden and more fragmented PRO networks.
- Fatigue, physical functioning, sexual problems, and work emerged as key bridge symptoms.
- Negative health outlook, distress, and physical functioning were core targets across groups.

Importance of the Study

Understanding the long-term functional impact of cancer during adolescence and young adulthood is critical, yet prior studies have evaluated patient-reported outcomes (PROs) mainly as isolated symptoms. By applying network analysis to more than 4000 adolescent and young adult (AYA) cancer survivors, this study provides a novel, systems-level view of how symptoms cluster and reinforce one another many years after diagnosis. Survivors of central nervous system (CNS) tumors showed not only higher symptom burden but also more diffuse and weakly integrated PRO networks, suggesting higher vulnerability but also potentially less cross-domain interventional effects. Several symptoms, fatigue, physical functioning, sexual concerns, and work limitations, acted as key bridge nodes though, which may represent key intervention targets. Importantly, negative health outlook, health distress, and physical functioning emerged as central concerns across all survivors. These findings highlight opportunities to personalize survivorship care and inform the design of targeted neurorehabilitation programs for AYA cancer survivors.

A brain tumor invading normal central nervous system tissue and its treatment can lead to brain dysfunction and related long-term sequelae.^1^^,^^2^ This is particularly concerning for adolescents and young adults (AYAs), 18 to 39 years at initial cancer diagnosis, whose brains are still in a critical stage of development.^3–5^ In addition, this period of life is marked by important milestones such as education, the pursuit of independence, employment, and an active lifestyle, all of which can be disrupted by the effects of the disease and its treatment.^6^^,^^7^ Depending on the age of the patient, brain tumor type, location and applied treatment, the neurological and neurotoxic impact, respectively, can lead to specific neurobehavioral outcomes.^8–11^

Increasing attention is given to potential long-term (neuro-)psychological and socioemotional symptoms in this AYA neuro-oncological population. These patients are known to be more susceptible to symptoms of depression,^12^ anxiety,^13^ and cognitive complaints related to attention, memory, and executive functioning,^14^ lower academic achievement, and decreased quality of life in general.^15–17^ To effectively prevent this and intervene early, it is crucial to identify individual risk factors and potential targets for intervention. Such risk factors include both patient-related and medical determinants, as well as physical and socio-emotional symptoms that may co-occur with, or contribute to, other complaints. In AYAs with brain tumors specifically, medical characteristics comprise the histological and molecular tumor features, tumor size and location, cranial radiation (volume and dose), (intraventricular) chemotherapy, and potential adverse events.^18–20^ Each of these parameters can induce or exacerbate the abovementioned psychological symptoms after treatment. Individual (coping strategies, resilience and cognitive reserves, age, sex)^21^ and contextual factors (socioeconomic position, family support, educational support, and access to healthcare)^15^^,^^22^^,^^23^ can further modulate these daily life outcomes.

To identify risk factors and tailor interventions that could minimize long-term psychological symptoms, novel approaches in investigating individual symptoms, risk factors, and their interdependencies are becoming crucial in personalized medicine. One of these approaches includes network analyses, which have increasingly received attention in the field of psychology.^24^^,^^25^ In this framework, symptoms and risk factors are modeled as a network, based on their covariances. This assists in determining individual features that are most central in the network, that is, that are closely related to a multitude of long-term issues, which is important in defining targets for prevention and intervention.

In the field of psycho-oncology specifically, network analyses have been applied to a limited extent. One of the initial studies by Schellekens et al. (2020) suggested fatigue to be a central complaint in psychological networks of non-CNS cancer patients.^26^ Another study in AYAs suggested clusters of daily life functioning, worriment, psychological, and sexual symptoms across a wide range of cancer diagnoses.^27^ However, large heterogeneity in disease mechanisms, symptoms, and impact exists between the different oncological subgroups. Brain tumors are associated with specific patient-related, tumor-related, and treatment-related factors, which need to be taken into account when profiling risk. In neuro-oncology, recent work has begun to characterize the co-occurrence and burden of symptoms at scale. Using large language models applied to electronic health records, Rhee et al demonstrated that patients with CNS tumors frequently experience multiple concurrent symptoms during therapy, with fatigue emerging as the most prevalent complaint.^28^ While this approach provides important insight into real-world symptom burden, it does not explicitly model symptom interdependencies or network structure.

To date, only a limited amount of studies have applied formal psychological symptom network modeling in adult neuro-oncology.^29^ Röttgering et al^29^ demonstrated that fatigued patients showed higher global strength in their symptom networks, suggesting high interdependency of symptoms in this subgroup. In addition, Bergsneider et al applied symptom network analysis in a large cohort of patients with primary brain tumors, identifying fatigue as a pivotal symptom and revealing distinct patient subgroups showing different dominant symptom profiles (eg, cognitive-driven burden in older patients).^30^ Still, due to different pathologies, treatments, and neuroplasticity windows, patients can be affected very differently. Furthermore, despite earlier research in neuro-oncology suggesting long-term psychological sequelae,^31^^,^^32^ the interdependencies between symptoms remain unknown, and risk factors have not yet been investigated from a network perspective. To date, symptoms have been mostly investigated as separate entities, rather than symptom clusters. Hence, it is important to identify and address the “core” symptoms and associated risks, to enable more timely interventions alleviating complaints and overall burden more profoundly in the future. Beyond symptom-focused approaches, network-based methodologies have also been explored to characterize social networks. One social network study showed that patients with glioblastomas reported relatively large and functionally differentiated social networks.^33^ Such findings underscore the relevance of network frameworks in this population, also outside the symptom domain.

In the current study, we applied a network analysis on a large database of AYA cancer survivors, comparing the psychological network topology of survivors of primary CNS tumors with those with non-CNS cancers. Within the CNS subgroup, we also explored the role of individual medical risk factors. These findings may contribute to further development and optimization of tailored neurorehabilitation programs in neuro-oncological care.

## Methods

### Participants

Based on the Netherlands Cancer Registry (NCR), which includes clinical data on all patients diagnosed with cancer in the Netherlands, participants were contacted if they had been diagnosed with cancer for the first time between 1999 and 2015, at the age of 18 to 39 years, and had received treatment in a university hospital or the National Cancer Institute in the Netherlands. Tumor types with extreme rarity or a favorable prognosis (eg, neuroendocrine tumors of the gastrointestinal tract, cancer of unknown primary site, skin adnexal carcinoma, unspecified skin carcinoma, squamous cell carcinoma of the skin, basal cell carcinoma, dermatofibrosarcoma, Kaposi sarcoma, atypical lipoma, atypical chondroma, placental trophoblast tumors, and cutaneous lymphomas), or patients who were not treated with curative intent, were excluded. In addition to these inclusion criteria, patients were eligible if they demonstrated sufficient proficiency in Dutch and adequate cognitive abilities to complete the questionnaires. The review board of the National Cancer Institute approved the study proposal (IRB-IRBd18122), and the study was registered as a clinical trial (NCT05379387).

### Sociodemographic and Medical Information

Based on a cross-sectional survey, sociodemographic data were collected, including the participants’ age at the time of questionnaire completion, sex, marital status, and educational level. In addition, clinical information was obtained from the NCR, including time since diagnosis, tumor histology, tumor location, tumor size, primary treatment (ie, chemotherapy yes/no, radiotherapy yes/no, hormone therapy yes/no, stem cell transplantation yes/no, surgery yes/no, awake surgery in case of a brain tumor), and genetic tumor profiling (if available). The International Classification of Diseases for Oncology (ICDO-3),^34^ and the TNM system or the Ann Arbor classification for Hodgkin and non-Hodgkin lymphoma,^35^ were applied for cancer type and stage categorization.

### Assessment of Health-Related Quality of Life

Survivors completed a patient-reported outcome (PRO) measurement about quality of life, lifestyle habits, and late effects. All data were collected between 2019 and 2021 and registered within the PROFILES (Patient-Reported Outcomes Following Initial treatment and Long-term Evaluation of Survivorship) registry.^36^

The EORTC QLQ-SURV100 is a 100-item inventory assessing long-term complaints in survivors, of which the items are addressed on a 4-point Likert scale from 1 (not applicable) to 4 (very much applicable). These items cover physical, mental, and social HRQoL. Based on the raw data, 33 PRO scores were calculated, including 13 functional scales (Physical Functioning, Role Functioning, Emotional Functioning, Cognitive Functioning, Sexual Functioning, Body Image, Symptom Awareness, Positive Health Behavior Change, Positive Life Outlook, Positive Impact on Behavior Toward Others, Positive Social Functioning, Work, and Global Health Status), 9 symptom scales (Pain, Fatigue, Sleep Problems, Social Interference, Social Isolation, Sexual Problems, Sexual Problems When Sexually Active, Health Distress, and Negative Health Outlook), and 11 single-item scores (Financial Difficulties, Treated Differently, Worrying of Cancer impact on children, Worry Cancer Risk, Fertility, Deeper Meaning, Problems Insurances loans mortgages, Worry Cancer Risk, Loss of Income, Partner Relation Stronger, Sexual Pleasure).

### Statistical Analyses

In the current study, we specifically compared PRO networks based on the scales derived from the EORTC QLQ-SURV100 questionnaire between survivors of CNS tumors (“CNS group”) and non-CNS cancers (“non-CNS group”). Before performing the network analyses, Wilcoxon rank-sum tests were used to test group differences in the original PRO scale scores (ie, level of complaints).

For consistency within the network analysis, both multi-item scales and single-item measures were treated as individual PRO nodes. When referring to specific QLQ-SURV100 scores, the term “scale” is used throughout to denote both multi-item scales and single-item measures. For network analyses, data processing was performed as follows. First, missing data were handled by recoding dataset-specific missing value codes (eg, 999 to NA). QLQ-SURV100 scale scores were then computed by averaging available items within each scale and applying standard linear transformations to a 0 to 100 scale, with reversed scoring where appropriate. To homogenize the scales across the variables included in our network analyses, all continuous PRO variables were linearly transformed to a standardized scale (ie, 0-100) and reversed when items were negatively phrased (ie, after transformation, higher scores consistently indicate better outcomes). No stochastic or multiple imputation was performed. Second, Gaussian graphical models (GGMs) were estimated as sparse partial correlation networks using graphical LASSO on the bivariate Spearman correlation matrix, applying an extended Bayesian information criterion tuning parameter of 0.25.^37^ Correlations were estimated using pairwise complete observations, to maximize data retention. Auto-correlations were retained. The resulting regularized inverse covariance matrix consisted of edges representing the partial correlations between variables, controlling for the remaining ones.

The organizational properties of these psychological symptom graphs were then statistically compared between the CNS and non-CNS groups using the permutation-based Network Comparison Test (NCT).^38^ Specifically, differences between the 2 groups in psychological network topology included the overall network strength, hub topology, and individual edge weights, based on 1000 permutations. Stability of edge weights was checked based on group-specific confidence intervals of permuted edge weights (ie, for the CNS group and non-CNS group). With the aim of detecting differences in group-specific hubs (ie, most central nodes), we focused on 4 centrality metrics: nodal strength and expected influence (EI), which indicate how strongly a symptom is connected to others within the network, and bridge strength and bridge EI, which show how much a symptom links different symptom domains. Strength takes both positive and negative edge weights into account, whereas EI considers only edges with the same sign.

Graph metrics that were significantly different between CNS versus non-CNS groups were further explored in smaller subgroups of CNS cancer survivors. Specifically, differences in PRO scales among these smaller groups were again assessed using the Wilcoxon rank-sum tests and group-based graph metrics were visually explored, that is, across treatment groups (eg, chemotherapy, cranial radiation, neurosurgery [yes vs no]), tumor locations, histology, and grades (for gliomas only). These subgroup comparisons were performed only when n > 25 per subgroup, and graph metrics were based on subgroup-specific bivariate correlation networks (for cross-group stability in graph metric estimations).

Finally, subgroup-specific networks (CNS vs non-CNS) were visualized using qgraph, with edges representing the partial correlation values (thickness of edges; positive edges in solid grey and negative edges as dashed grey lines); nodes were scaled by their nodal strength and were color-coded and spatially ordered according to their community membership (using Louvain clustering).^39^

## Results

In total, 4005 AYA survivors participated, of whom 164 were survivors of primary CNS tumors and 3841 had non-CNS cancers.

Median age at diagnosis was 31 years (SD = 6.1) in the CNS group and 33 years (SD = 5.9) in the non-CNS group. A higher proportion of CNS survivors received radiotherapy (51% vs 32%) and surgery (80% vs 78%), whereas chemotherapy was more common in the non-CNS group (57% vs 21%). Additional demographic and clinical characteristics are summarized in Table 1.

**Table 1.** Demographic information of patient population

Characteristics	CNS group (*n* = 164)	Non-CNS group (*n* = 3841)	All (*n* = 4005)
**Demographic**			
Median age at incidence (SD)	31 (6.1)	33 (5.9)	33 (5.9)
Gender: Females	79	2376	2455
**Chemotherapy**			
No	130	1637	1767
Yes	34	2204	2238
**RT**			
No	80	2023	2103
Yes	84	1818	1902
**Surgery**			
No	32	840	872
Yes	132	2978	3110
**Hormone therapy**			
No	164	3357	3521
Yes	0	484	484
**Stem cell transplantation**			
No	164	3700	3864
Yes	0	141	141
**Education**			
Primary school or equivalent	1	27	28
Secondary school or equivalent	76	1644	1720
College/University or equivalent	87	2162	2249
**WHO grade**			
Grade 1/well differentiated	5	182	187
Grade 2/moderately differentiated	77	513	590
Grade 3/poorly differentiated	28	652	680
Grade 4/undifferentiated/anaplastic/GGG4	23	14	37
T cell/GGG5	NA	39	39
B cell	NA	181	181
Unknown	31	2260	2291
**Tumor histology**	Astrocytomas Glioblastomas Ependymomas Medulloblastomas Oligodendrogliomas Pineoblastomas/PPTID Gangliogliomas Chordomas Meningiomas Schwannomas (eg, acoustic neuroma) PNETs/embryonal CNS tumors Granular cell tumors	Breast GI tract (eg, colon, stomach, esophagus) Lung Kidney Liver Pancreas Skin (eg, melanomas) Hematological malignancies (eg, leukemia, lymphoma) Endocrine organs (eg, thyroid, adrenal) Urogenital system (eg, ovaries, testis, prostate, uterus) Soft tissue/osteo-sarcomas Extracranial germ cell tumors	

Abbreviations: CNS, central nervous system; GI, gastrointestinal; PNETs, primitive neuroectodermal tumors; PPTID, pineal parenchymal tumor of intermediate differentiation; RT, radiation therapy; WHO, World Health Organization.

**Figure 1.**
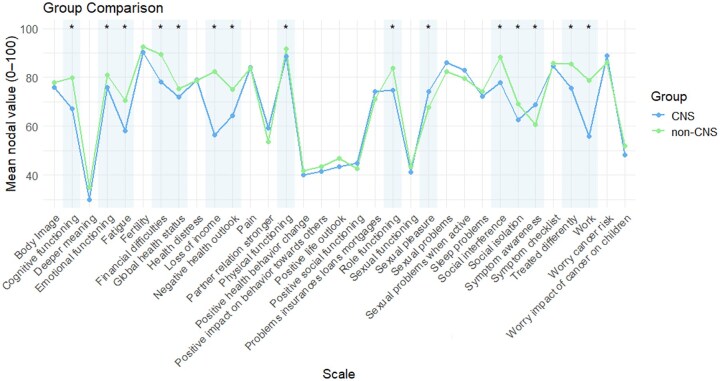
Group differences (CNS vs non-CNS cancer survivors) in PRO scores on the SURV100. Group average scores are indicated in green and blue, for the non-CNS versus CNS group, respectively. All PRO scales were scaled with a value between 0 and 100, where a higher score indicates better functioning. Significantly different scales are indicated with a star, based on Wilcoxon rank-sum group comparisons (*P* < .05). For detailed statistics, see Supplementary Materials (Table S2). Abbreviations: CNS, central nervous system; PRO, patient-reported outcomes.

Based on the Wilcoxon rank-sum tests, significant differences between CNS and non-CNS groups were found in 15 of the 33 scales (Figure 1). Specifically, on these significant scales, the CNS group scored lower (ie, reported more complaints) than the non-CNS group, except for Sexual Pleasure and Symptom Awareness (where the effect was reversed). The largest differences among these scales were observed in socially related outcomes (ie, Financial Difficulties, Loss of Income, Role Functioning, Social Interference, Work), Cognitive Functioning, and Fatigue.

**Figure 2.**
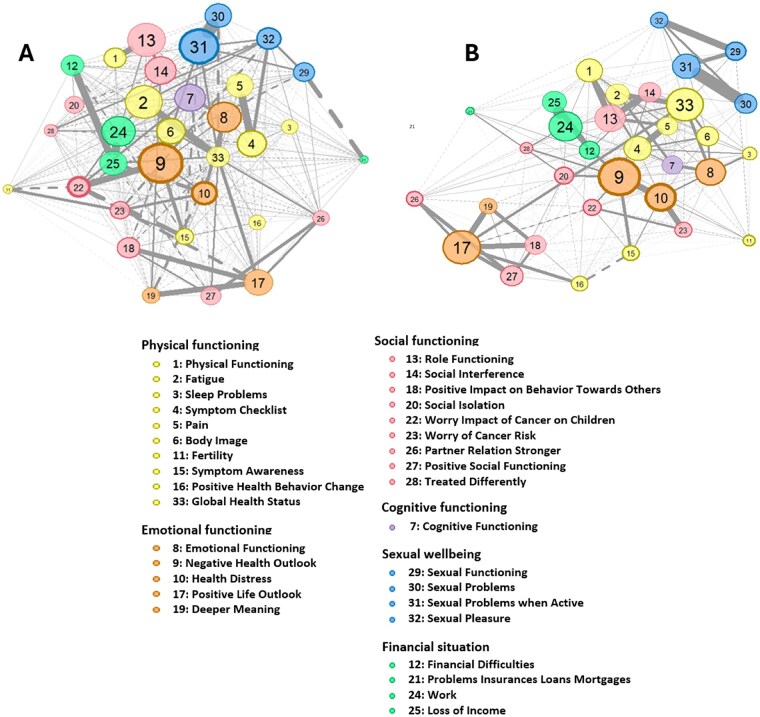
Subgroup-specific PRO networks. Panel A presents the average PRO network of CNS survivors. Panel B depicts the average network of the non-CNS cancer survivors. In this figure, nodes are colored according to the original PRO scales. Nodes are positioned according to community membership per subgroup. Edge thickness reflects the magnitude of partial correlations. Positive correlations are shown with grey edges. Negative correlations are represented by dashed edges. Abbreviations: CNS, central nervous system; PRO, patient-reported outcomes.

When investigating the PRO networks, the confidence intervals of estimated edge weights demonstrate high variability in the CNS population, compared with the non-CNS group (Figure S1). Group-specific PRO networks are shown in Figure 2.

**Figure 3.**
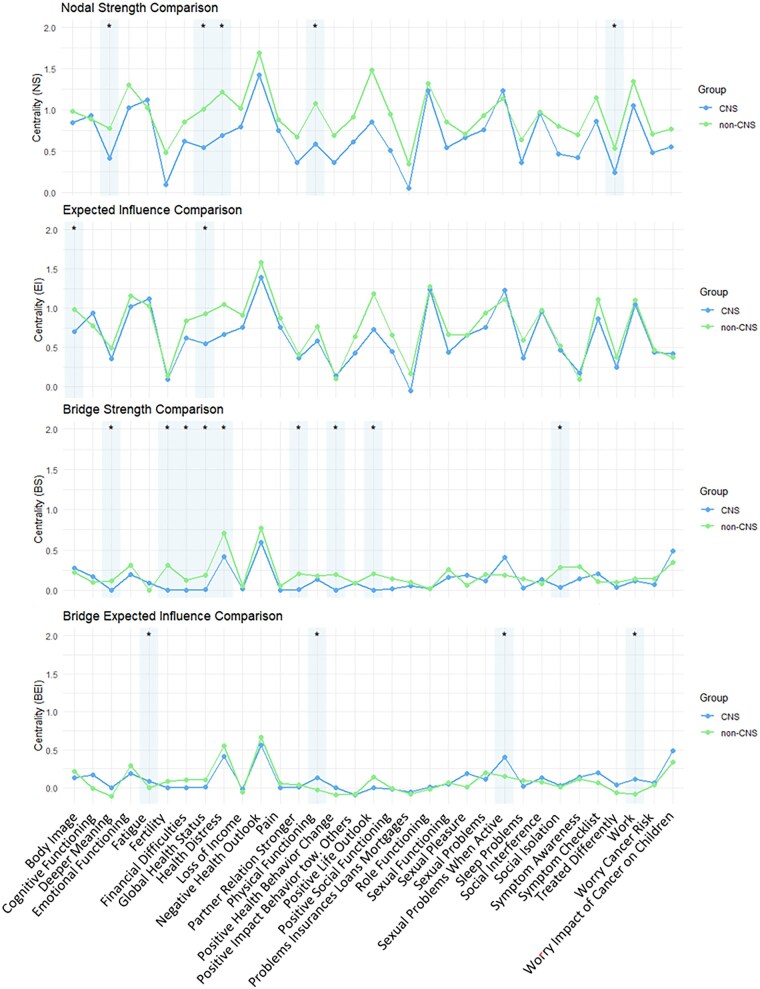
Group differences in nodal centrality metrics across PRO scales. Group-specific graph metrics are indicated in green and blue, for the non-CNS versus CNS group, respectively. Significantly different metrics are indicated with a star, based on the NCT group comparisons (*P* < .05). Abbreviations: CNS, central nervous system; PRO, patient-reported outcomes.

Global strength across the entire network was significantly different between groups (*P* = .0360). However, the individual nodes appeared to exhibit a higher nodal strength across most scales in the non-CNS compared with CNS group (Figure 3A). Although the CNS group demonstrated a higher number of edges (after regularization), as can be seen in Figure 2A, their sums per node (ie, nodal strength) were mostly lower than in the non-CNS group, indicating weaker connections in the CNS group.

These group differences were significant for several nodes. More specifically, higher nodal strength was found in the non-CNS group for the scales Deeper Meaning (*P* = .0366), Global Health Status (*P* = .0008), Health Distress (*P* = .0343), Physical Functioning (*P* = .0060) and Treated Differently (*P* = .0240) (Figure 3). Bridge strength was also significantly higher in the non-CNS group for Deeper Meaning (*P* = .0394), Fertility (.0004), Financial Difficulties (*P* = .0140), Global Health Status (*P* = .0020), Health Distress (*P* = .0234), Partner Relation Stronger (*P* = .0324), Positive Health Behavior Change (*P* = .0034), Positive Life Outlook (*P* = .0056), and Social Isolation (*P* = .0148). Regarding EI, this metric was significantly higher in the non-CNS group for Body Image (*P* = .0382) and Global Health Status (*P* = .0110). In other words, these PROs appear to be significantly more central within the network of non-CNS cancer survivors. By contrast, higher bridge EI was found in CNS cancer survivors for Fatigue (*P* = .0278), Physical Functioning (*P* = .0080), Sexual Problems When Active (*P* = .0066), and Work (*P* = .0232). Reflecting how much a node connects different communities, higher bridge EI suggests a key link between domains, such as social experiences and emotional outcomes in the CNS group.

**Figure 4.**
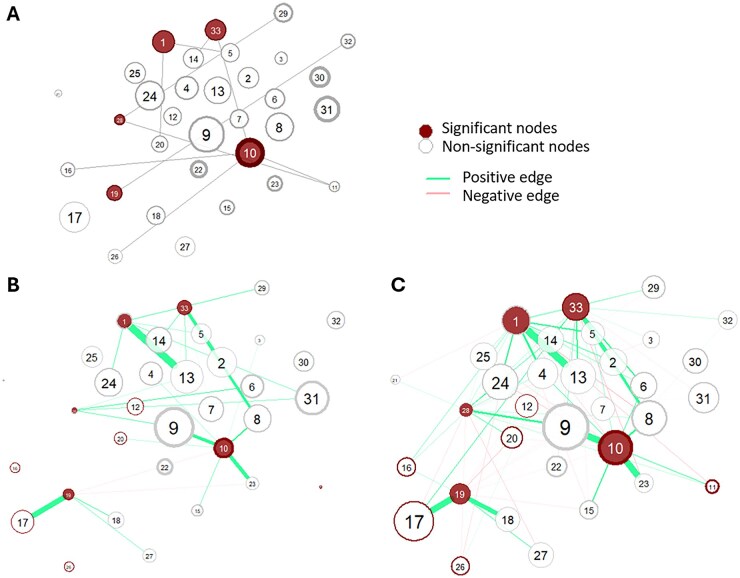
Networks presenting significant group differences in edge weights and nodal strength. Panel A presents the significantly different edges of the nodes with significantly different nodal strength. In panel B, the edges of these nodes in CNS cancer survivors are presented. In panel C, the edges of these nodes in the non-CNS cancer survivors are presented. Nodal sizes are according to nodal strength per subgroup. Nodes in red indicate the significantly different nodes (in nodal strength). Nodal locations are according to community assignment for the overall group. Abbreviation: CNS, central nervous system.

In addition, specific edges showed significant differences in edge weights, suggesting that certain associations may be population-specific (Figure 4). Specifically, of the nodes presenting higher strength in non-CNS cancer survivors, the majority had multiple strong connections that were absent or weaker in the CNS group.

**Figure 5.**
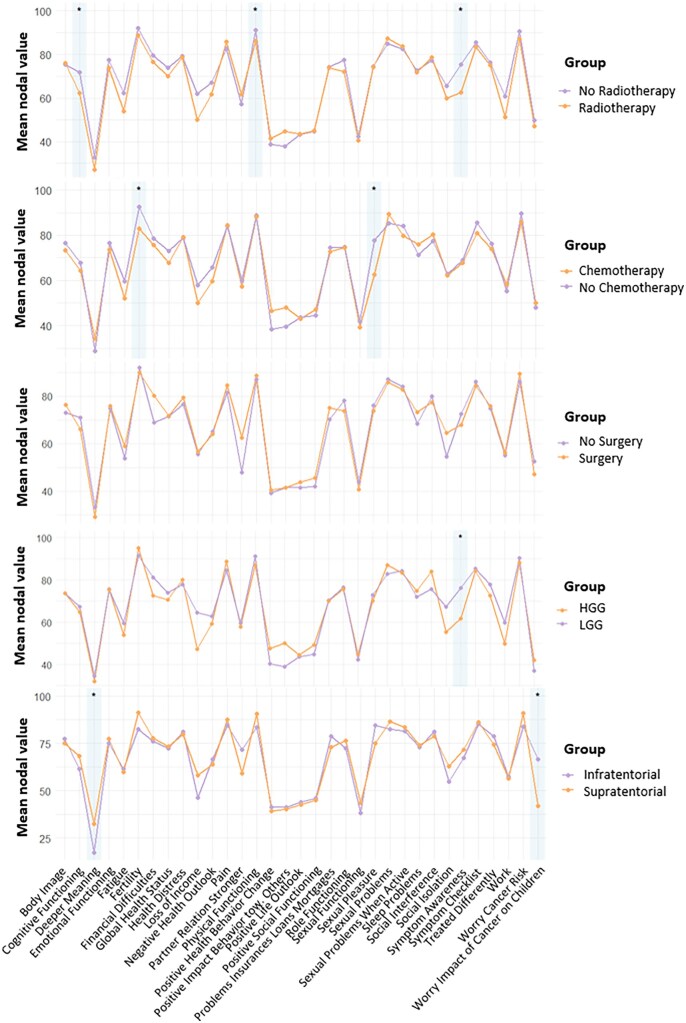
PRO scale subgroup differences within the CNS group. Based on Wilcoxon rank-sum tests, subgroup comparisons in PRO scale values were tested within the CNS group (based on RT vs no RT, chemotherapy vs no chemotherapy, infra- vs supratentorial tumors, high-grade vs low-grade gliomas [within glioma group only]). Blue bars with a star indicate significant subgroup differences (among the CNS subgroups). Abbreviations: CNS, central nervous system; PRO, patient-reported outcomes; RT, radiation therapy.

When focusing on risk stratification within the CNS population, data were sufficient to exploratorily and descriptively examine differences in PRO scales and their network features with regard to tumor grade (high-grade vs low-grade gliomas; n = 31 vs n = 59) and tumor locations (supratentorial vs infratentorial tumors; n = 99 vs n = 25) (see Table S1; Figure 5 and Figure S3, for PRO scales and network features, respectively). These subgroup analyses were conducted to illustrate potential patterns within the CNS group and were not intended for definitive inference. Regarding treatment effects, the application of cranial radiation, chemotherapy, and surgery was explored. First, regarding the raw PRO scale values, survivors who received irradiation showed more difficulties with Physical functioning, higher Symptom Awareness and Cognitive Functioning (*P* = .0390, *P* = .0006, *P* = .0482, respectively) (see Figure 5). Survivors treated with chemotherapy had more complaints with regard to Fertility and Sexual pleasure (*P* = .0434, *P* = .0343, respectively). Surgery (yes vs. no) did not yield significant effects on scale scores in this cohort. Survivors of supratentorial tumors showed more worrying about the impact of the disease on their children (*P* = .0239), while survivors of infratentorial tumors showed lower levels of Deeper meaning (*P* = .0423). It should be noted that infratentorial tumors in this cohort were predominantly embryonal, limiting the ability to disentangle effects of tumor location from tumor histology. Within the glioma subgroup, survivors of the high-grade gliomas demonstrated higher levels of symptom awareness (*P* = .0056). Regarding the PRO network properties, differences between CNS groups could only be visually explored due to limited power. Observed subgroup patterns should therefore be interpreted cautiously. Some earlier observed differences in the CNS versus non-CNS group may have been partly driven by (or are more notable in) certain CNS subgroups. Specifically, effects found in lower EI might be more pronounced in survivors who received chemotherapy, had supratentorial and high-grade gliomas, the lower bridge strength in survivors of supratentorial tumors, and the higher bridge EI in patients with infratentorial tumors (see Figure S2).

## Discussion

This study investigated PRO networks in AYAs. At the symptom level, CNS cancer survivors reported a greater overall burden than non-CNS cancer survivors on most outcomes. The largest effects were observed in social outcomes (Financial Difficulties, Loss of Income, Role Functioning, Social Interference, Work), Cognitive Functioning, and Fatigue. By contrast, Sexual Pleasure and Symptom Awareness were lower in the non-CNS group, plausibly reflecting systemic/endocrine treatment effects or differences in health monitoring or behavior. Network analyses provided additional insights by identifying a few PROs that showed stronger cross-connections between different parts of the network in CNS cancer survivors, suggesting a small set of “gateways” (Fatigue, Physical Functioning, Sexual Problems When Active and Work), through which difficulties may spread, even though their overall centrality is lower. By contrast, multiple nodes appeared as more central in the non-CNS group, particularly in outcomes related to health and social functioning or meaning making.

Our findings suggest that CNS cancer survivors carry a higher symptom burden across multiple PRO scores, whereas in non-CNS cancer survivors PRO constructs appear more embedded and cross-linked across clusters. Regarding the overall burden, the elevated difficulties in social outcomes (related to work, finances and social interference),^40^^,^^41^ as well as cognitive^42^ and fatigue outcomes, in the CNS group are in line with previous research.^43^ Moreover, previous studies in CNS cancer patients have shown that lower cognitive functioning can significantly affect overall quality of life,^44^ but that this link may be obscured in patients with cognitive impairment.^45^ In addition, it was previously shown that fatigue and cognitive impairment are not necessarily linked and, therefore, require independent and complementary assessment.^46^ In this regard, cognitive decline or deficits in CNS tumor survivors may differently affect how symptoms are perceived and interpreted, and consequently how they are related to one another.^3^^,^^45^^,^^47^ The higher overall symptom burden reported by CNS survivors together with higher self-reported symptom awareness may therefore partly reflect differences in cognitive processes underlying symptom interpretation. In the same vein, indirect effects of altered cognitive functioning may also contribute to the more diffuse symptom networks observed. Future studies incorporating objective cognitive measures are therefore recommended to disentangle such potential meta-cognitive influences among psychological outcomes.

Based on the network analyses, the CNS group showed a higher number of edges in their PRO networks, which were less organized, and nodal strengths were consistently lower across nearly all PROs. This suggests that although more symptoms and more associations exist in this population, they are generally weaker than in non-CNS survivors. In other words, while the non-CNS group demonstrated a more cohesive QoL-meaning-making positive-adaptation core (higher strength, EI and bridging across many nodes) with modularity, the CNS group showed a more diffuse (or less modular), poorly integrated PRO network. This observation may reflect disease-related factors, including neurobiological or cognitive constraints that affect the typical organization of psychological networks. In addition, the abovementioned studies demonstrated possible effects of objective cognitive functioning on PRO responses and possible answer biases,^45^^,^^47^ which could explain higher within-CNS-group heterogeneity (cfr. grade effects infra) and reduced organization in the observed networks. Their lower nodal strength further suggests a more fragmented psychosocial profile.

Additionally, reduced bridge strength in some nodes supports the idea that cross-domain integration, between emotional, cognitive, physical, and social domains, is limited in the CNS population. Although the CNS group showed fewer or weaker nodes acting as bridges across diverse domains, connections of nodes that did function as such (eg, Fatigue, Physical functioning, Sexual problems, Work) were consistently signed, resulting in higher bridge EI. This suggests these nodes may act as key bridge symptoms connecting different symptom domains. As bridge EI is sensitive to the direction of connections, even a small set of strong, same-direction cross-domain links can increase bridge EI, even if total cross-domain connectivity (bridge strength) is low. Clinically, this supports the idea that in CNS cancer survivors these nodes could be the main conduits through which difficulties spread across domains of physical, emotional, and social functioning. Targeting these nodes may yield network-wide downstream effects despite lower global centrality elsewhere. The fact that the CNS group reported both higher levels and higher bridge EI of fatigue suggests it may be an important gateway. In relation to previous network analyses in neuro-oncology, where fatigued patients showed higher network strength (ie, more coherence between symptoms),^29^^,^^30^ and clustering approaches have demonstrated clusters of symptoms related to motor and fatigue symptoms in CNS cancer patients,^48^ our findings provide additional information on the bridging role of fatigue between domains. Moreover, previous research has shown that fatigue affects the ability to return to work,^40^ where we show both factors to be inter-domain links. Similarly, sexual problems are relatively common in this population and important to address.^49^

By contrast, the non-CNS group showed higher centrality of multiple health, social, and meaning-making nodes. These findings suggest that in the non-CNS group multiple symptoms might serve as key hubs linking domains, whereas CNS networks rely on only a few functional bridges. Similar PRO network studies in non-CNS cancer populations likewise identified fatigue, depression/emotional distress, sleep problems, negative health outlook/global health, and coping processes as central nodes, although often assessed with diverse graph metrics across studies. Together, these findings suggest a more cohesive, hub-driven structure in these groups.^27^^,^^50–52^

Abovementioned topological differences in PRO networks may explain why similar symptoms propagate differently across groups and suggest tailored intervention targets. In non-CNS cancer survivors, global/meaning-making and positive adaptation nodes are both central and bridging. Targeting these nodes may yield broad, network-wide benefits. In CNS cancer survivors, combining the information on their complaint levels with network centrality suggests fatigue management, physical exercise/rehabilitation, vocational support, and targeted sexual counseling as leverage points that may yield cross-domain benefits due to higher bridge EI. Evidence from small intervention trials in neuro-oncology indicates that physical rehabilitation is feasible and could improve both physical functioning,^53^ fatigue,^54^ and cognitive outcomes in glioma.^55^ Vocational rehabilitation programs can additionally facilitate return-to-work.^56^ Beyond such focused interventions, the identification of weaker, less centralized networks in the CNS group underscores the importance of multimodal support strategies, as single-domain interventions may have less potential to cascade. By contrast, social or emotional interventions may be effective in non-CNS cancer populations, with cross-domain generalization. Importantly, in both groups Negative Health Outlook, Health Distress and Physical Functioning appeared consistently central across all 4 graph metrics. These health-related concerns are crucial to target in both populations.

When investigating CNS subgroups in more detail, radiotherapy-treated patients reported more difficulties in Physical Functioning, Symptom Awareness, and Cognitive Functioning. As discussed earlier, these bridging nodes are important intervention targets in CNS cancer survivors. Prior studies also reported post-RT worsening in physical functioning as well as fatigue,^57^^,^^58^ especially in the first few months after treatment, with longer-term HRQoL deterioration and dose/volume–related effects on role/cognitive functioning.^59^^,^^60^ Furthermore, survivors who received chemotherapy reported more concerns regarding Fertility and lower Sexual Pleasure. Such (neo-)adjuvant treatment places a burden on the whole body, explaining the increased side effects. Survivors with supratentorial tumors worried more about the impact on children, whereas those with infratentorial tumors reported less Deeper Meaning. However, given that infratentorial tumors in this cohort were largely embryonal, these findings likely reflect a combination of tumor location, histology, and age-related or developmental factors, rather than location alone. Tumor location effects could be partly explained by different functional units or networks in the brain being affected,^18^^,^^61^^,^^62^ but also different cognitive consequences associated with tumor type or location could lead to different perspectives on these PROs.^63^^,^^64^ Finally, higher Symptom Awareness in high-grade compared with low-grade gliomas aligns with evidence that high-grade glioma patients experience greater overall symptom burden and poorer HRQoL.^45^^,^^65–67^ Tumor aggressiveness, treatment intensity (chemoradiotherapy with temozolomide) and prognosis-related distress may jointly contribute to these differences.

Although network metrics did not show consistent subgroup effects within CNS groups, exploratory trends toward lower EI appeared more pronounced with chemotherapy, supratentorial tumor location, and high-grade gliomas. Similarly, lower bridge strength was observed in supratentorial tumors, while higher bridge EI was seen in infratentorial tumors. Although these findings align with treatment and lesion factors (location, grade), given the small and heterogeneous subgroup sizes and the confounding between tumor location and histology, these network-level findings should be interpreted as hypothesis-generating rather than confirmatory. Larger, histologically stratified and longitudinal cohorts are required to more definitively assess such subgroup-specific network differences in more detail.

Several methodological considerations should be acknowledged when interpreting these findings. First, although we implemented regularization and permutation testing with partial correlation networks, alternative approaches may yield different network structures or centrality rankings (eg, cluster-based methods, latent covariance modeling, or Bayesian estimations). The choice of estimator and regularization parameters can substantially influence network topology, particularly in smaller or noisier datasets. Hence, we applied regularization in the CNS versus non-CNS comparisons, while for CNS subgroups we relied on visual inspection of metrics from nonregularized networks. Future work should assess the robustness of the CNS versus non-CNS network findings using larger databases of CNS cancer survivors. Similarly, the relatively small CNS sample introduces constraints. Although scale scores were calculated based on available items, without enforcing minimum item completion thresholds, which maximizes data retention, these scores may be less precise in respondents with higher item-level missingness. Bootstrapped confidence intervals around edge weights were wider in the CNS network compared with the non-CNS network, reflecting lower statistical power and increased variability in edge estimation. Regularization in this case may have introduced bias toward sparsity, potentially removing subtle but meaningful connections. As a result, true group differences may be underestimated. Although we used many bootstrap iterations (*n* = 5000) to enhance stability and precision, the inherent limitations of a small sample cannot be excluded. Still, in oncology, the inclusion of both CNS and non-CNS AYA cancer survivors (*n* = 4005) is exceptional. Second, the cross-sectional design precludes conclusions about temporal dynamics or network stability. Symptom networks can change over time, particularly as cancer survivors’ medical and psychosocial experiences shift across survivorship stages. It remains unclear whether observed group differences reflect stable trait-like patterns or transient state-related configurations. Longitudinal network analyses are needed to assess within-person dynamics and the temporal unfolding of symptoms. For instance, earlier work in CNS cancer survivors demonstrated that baseline depression and energy levels predicted depressive symptoms 1 year post-surgery.^68^ It is important to note that effects in such longitudinal studies likely differ between group-based changes and individual patterns.^69^  Third, graph theory allows derivation of many different graph metrics. In the current study, we focused on nodal features, including nodal strength, bridge strength, EI, and bridge EI. These metrics were selected as robust measures of centrality in sparse networks, capturing distinct aspects of symptom coherence across subgroups. This approach enabled identification of both within-domain and domain-bridging symptoms that may serve as key clinical targets for intervention. Finally, the population of CNS cancer survivors in this cohort is heterogeneous and included patients were diagnosed between 1999 and 2015. During this period, tumor classification was based exclusively on histopathology, mostly in line with the WHO CNS 2007 classification. Molecular markers such as IDH mutation, 1p/19q co-deletion, and other genomic alterations—now central to CNS tumor classification—were only introduced in the WHO CNS 2016 update and were not available for this study. Tumors were coded using ICD-O-3 morphology codes, adopted in the NCR from 2000. Consequently, tumor grading (eg, WHO grades I-IV) reflects histology only, and many low-grade gliomas likely remain of unknown molecular subtype. This limitation reflects the historical timeframe of diagnosis and reduces the granularity of CNS subtype analyses. While this classification approach aligned with clinical standards of the time, it does not fully capture the biological heterogeneity now recognized within CNS tumor types. Finally, we investigated potential effects of tumor-related and treatment-related risk factors in the CNS group, but were limited in scope due to small sample sizes of CNS subgroups. Furthermore, the clinical heterogeneity in the CNS group may contribute to greater variability in covariances across individuals, resulting in less stable networks. Consequently, group-level network differences should be interpreted as reflecting both true differences in symptom interrelations and effects of intrinsic clinical variability.

## Conclusion

In this large cohort of AYA cancer survivors, we found differences in both symptom burden and PRO network topology between CNS and non-CNS oncological populations. The CNS cancer survivors showed a higher overall burden, particularly in social/role participation, cognition, and fatigue, and more diffuse and more weakly integrated networks. Weaker within-domain symptom cohesion and limited cross-domain integration suggest that interventions in this population may propagate less readily, except through a few key role-limiting nodes (related to fatigue, physical functioning, sexual problems, work), which could act as cross-domain “gateways.” In CNS subgroups, symptom patterns are aligned with treatment and tumor factors, where radiotherapy and chemotherapy could yield more symptoms, tumor location influences PROs and network topology, and higher grade gliomas are associated with greater symptom burden. In contrast, non-CNS cancer survivors show a more cohesive, hub-driven architecture centered on health, social, and meaning-making nodes. Clinically, interventions for non-CNS cancer survivors may therefore focus on global and adaptive hubs, while CNS cancer care should prioritize gateway symptoms such as fatigue, physical rehabilitation, vocational support, and sexual counseling. Across both groups, negative health outlook, health distress, and physical functioning emerged as consistent core targets, warranting prioritization in follow-up and care for all cancer survivors.

## Supplementary Material

npag015_Supplementary_Data

## Data Availability

The data are not publicly available due to the local GDPR regulations and approval of the local ethical committee to use the data only locally. After data-transfer agreements and additional approval of the local ethical committee, pseudonymized data could only be shared upon request.
