# Supplementary material for: Symptom burden in young adult brain tumor survivors: Key intervention targets based on patient-reported outcome network analyses
Source: Neurooncol Pract. 2026 Feb 25;13(4):782–96. doi: 10.1093/nop/npag015 (PMC13365141; doi:10.1093/nop/npag015)

**Supplementary materials**

Figure S1. Bootstrapped edge weights for CNS and non-CNS tumor survivors


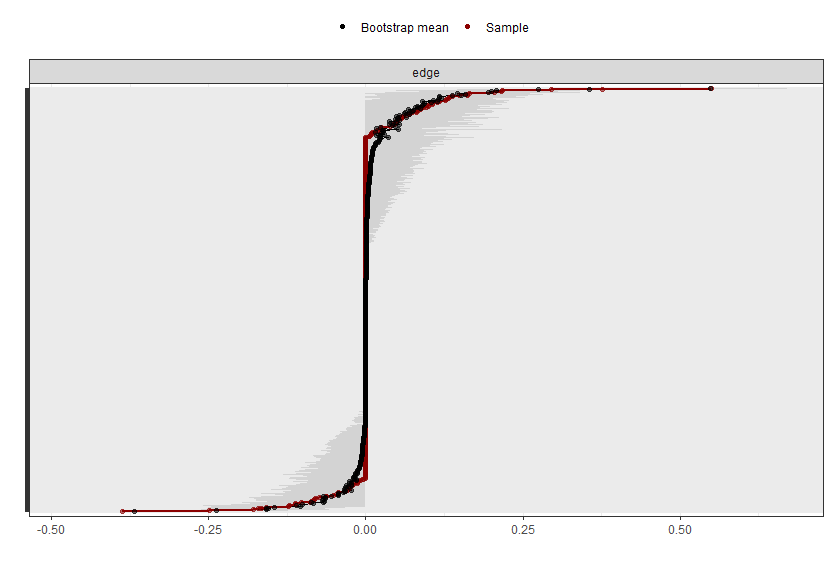

Note. Gray bands indicate 95% confidence intervals from bootstrapping. Black dots indicate the bootstrap means for edge weights. The red line indicates the original sample edge weights (sorted). Upper panel demonstrates figure for CNS tumor survivors, while lower panel provides the estimates for the non-CNS tumor survivors. As can be observed, confidence intervals are wider in the CNS survivor group. Bootstrapped means are close to the original values, suggesting stable edge estimates.


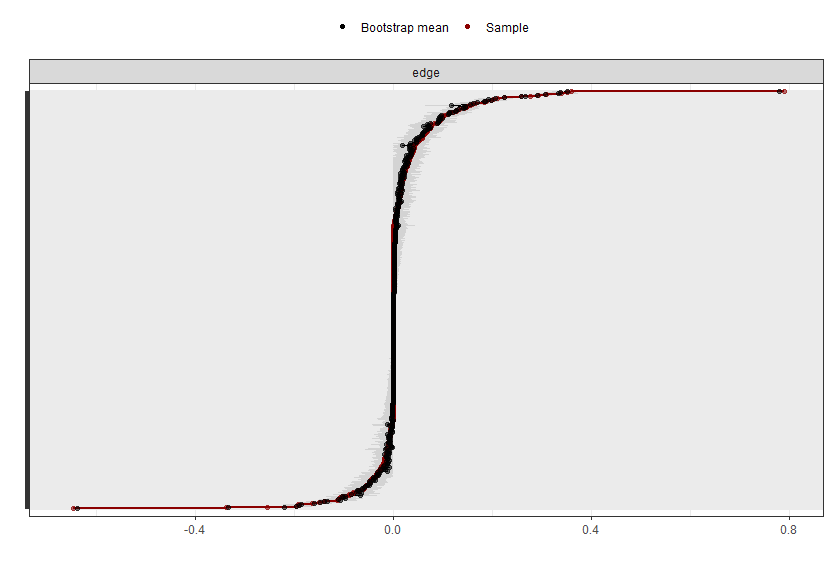


Table S1. Frequency table of CNS tumor histology and locations

|  | Infratentorial | Supratentorial | Pineal | Pituitary | Spinal | Meningeal | Other | Total |
| --- | --- | --- | --- | --- | --- | --- | --- | --- |
| HGG | 1 | 27 | 0 | 0 | 0 | 0 | 3 | 31 |
| LGG | 0 | 50 | 0 | 0 | 0 | 0 | 9 | 59 |
| Embryonal | 17 | 0 | 0 | 0 | 0 | 0 | 0 | 17 |
| Ependymal | 7 | 3 | 0 | 0 | 11 | 0 | 1 | 22 |
| Germ cell | 0 | 3 | 8 | 1 | 0 | 0 | 2 | 14 |
| Meningioma | 0 | 0 | 0 | 0 | 0 | 1 | 0 | 1 |
| Pineal | 0 | 0 | 2 | 0 | 0 | 0 | 0 | 2 |
| Schwannoma | 0 | 0 | 0 | 0 | 0 | 0 | 1 | 1 |
| Other | 0 | 16 | 0 | 0 | 0 | 0 | 1 | 17 |
| Total | 25 | 99 | 10 | 1 | 11 | 1 | 17 | 164 |

Note. * indicates a sample size of the subgroup reaching n>25 for group comparisons. The subgroups of high vs. low-grade gliomas and supra- vs. infratentorial tumors were compared. HGG = High-grade glioma, LGG = Low-grade glioma.

Table S2. Kruskall-Wallis test results comparing PRO scales between CNS and non-CNS tumor survivors

| SURV100 scale | Mean CNS group (n=164) | Mean non-CNS group (n=3841) | W | p |
| --- | --- | --- | --- | --- |
| Physical functioning | 88.485 | 91.597 | 306522.0 | 0.010 |
| Fatigue | 41.991 | 29.503 | 203148.0 | <0.001 |
| Sleep_problems | 27.706 | 25.708 | 255151.0 | 0.111 |
| Symptom_checklist | 15.436 | 14.374 | 258910.5 | 0.189 |
| Pain | 15.909 | 16.188 | 279336.0 | 0.777 |
| Body_Image | 75.758 | 77.731 | 284829.0 | 0.451 |
| Cognitive_functioning | 66.991 | 79.799 | 362241.0 | <.001 |
| Emotional_functioning | 75.755 | 80.905 | 306937.0 | 0.010 |
| Negative_health_outlook | 35.722 | 24.875 | 195023.0 | <0.001 |
| Health_distress | 21.060 | 21.215 | 271873.5 | 0.904 |
| Fertility | 9.649 | 7.380 | 258092.0 | 0.091 |
| Financial_difficulties | 22.004 | 10.579 | 219231.0 | <.001 |
| Role_functioning | 74.728 | 83.896 | 342060.5 | <.001 |
| Social_interference | 22.039 | 11.600 | 210529.5 | <.001 |
| Symptom_awareness | 31.250 | 39.475 | 319990.0 | <.001 |
| Positive_health_behavior_change | 40.132 | 41.741 | 283611.0 | 0.345 |
| Positive_life_outlook | 43.366 | 46.861 | 294253.5 | 0.079 |
| Positive_impact_on_behavior_towards_others | 41.338 | 43.412 | 282481.0 | 0.386 |
| Deeper_meaning | 30.000 | 34.930 | 291320.0 | 0.056 |
| Social_isolation | 37.390 | 30.800 | 235917.0 | 0.005 |
| Problems_insurances_loans_mortgages | 25.877 | 28.889 | 287108.0 | 0.187 |
| Worry_impact_of_cancer_on_children | 80.702 | 71.459 | 242407.0 | 0.023 |
| Work | 42.873 | 67.982 | 372496.0 | <.001 |
| Loss_of_income | 55.921 | 28.784 | 184085.0 | <.001 |
| Partner_relation_stronger | 82.340 | 71.322 | 232619.0 | 0.004 |
| Positive_social_functioning | 44.956 | 42.502 | 256959.0 | 0.256 |
| Treated_differently | 24.342 | 14.448 | 211129.0 | <.001 |
| Sexual_functioning | 50.109 | 48.159 | 267398.0 | 0.671 |
| Sexual_problems | 37.800 | 31.679 | 263272.5 | 0.433 |
| Sexual_problems_when_active | 49.891 | 40.074 | 251092.5 | 0.084 |
| Sexual_pleasure | 98.026 | 84.597 | 226085.0 | <.001 |
| Global_health_status | 71.786 | 75.383 | 311930.0 | 0.002 |

Figure S2. Nodal differences in centrality between CNS subgroups


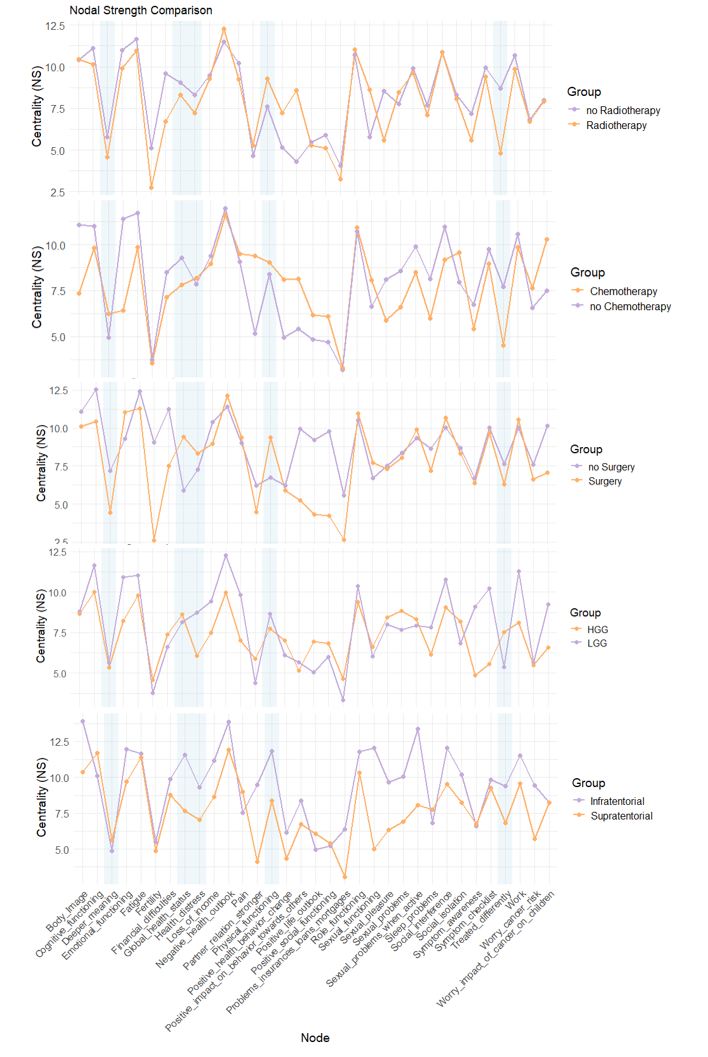


Note. Based on the NCT test, CNS vs. non-CNS groups were compared in graph metrics. Blue bars with a star indicate the significant group differences in these metrics (in CNS vs nonCNS). CNS subgroups were too small for NCT permutation testing, so can only be compared visually (purple vs. orange subgroups). Visual comparisons can be made here in graph metrics among the CNS subgroup (based on RT vs no RT, chemotherapy vs. no chemotherapy, infra- vs. supratentorial tumors, high-grade vs. low-grade gliomas (within glioma group only)).


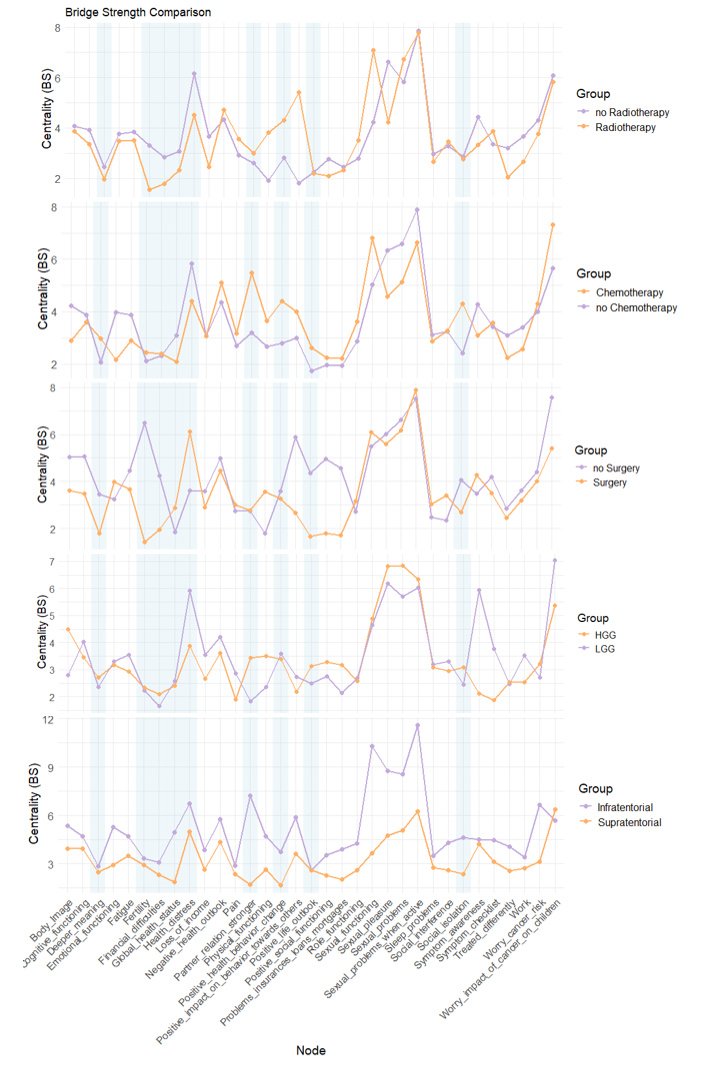


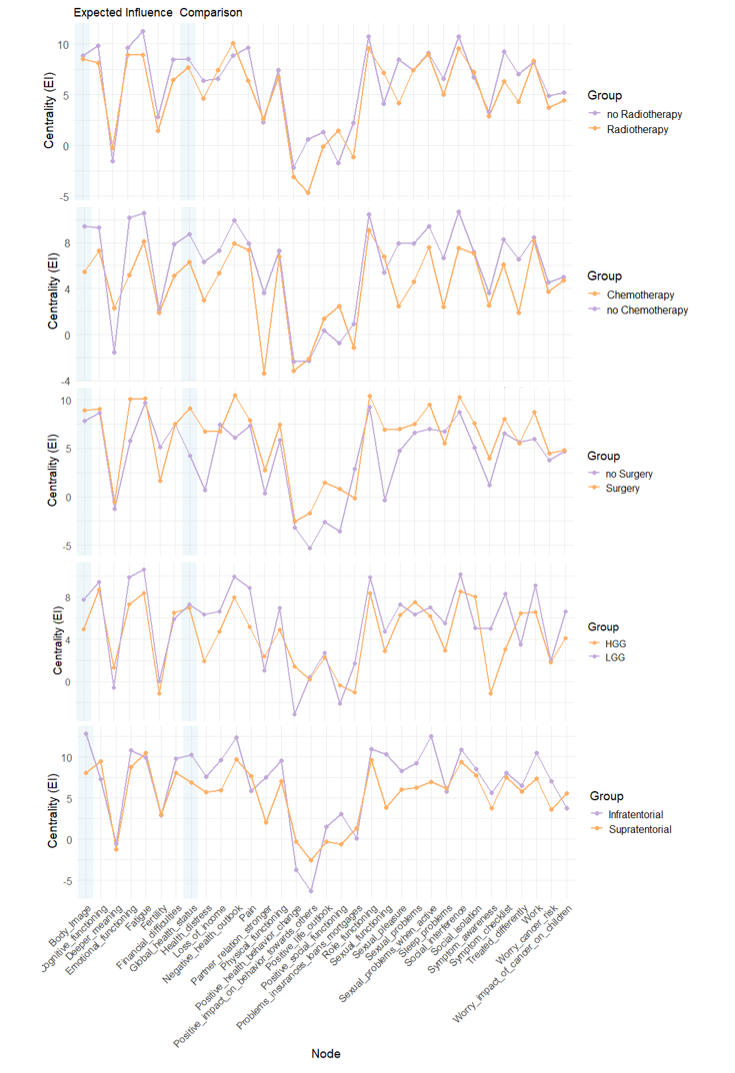


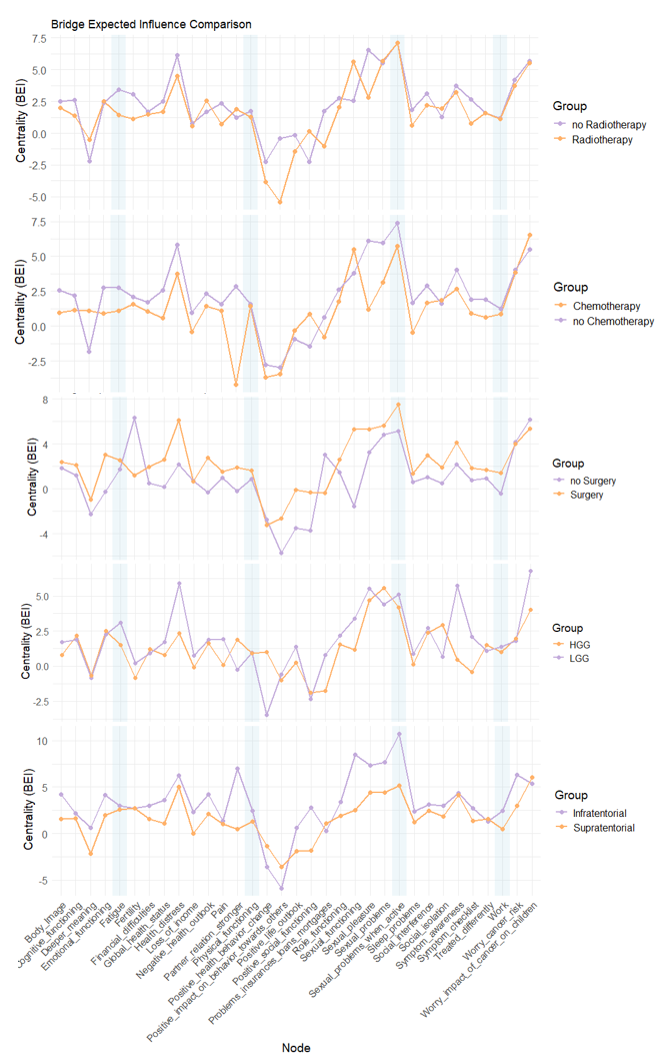

Supplement: npag015_Supplementary_Data [file npag015_supplementary_data.docx]
